# Polydatin Reduces Cardiotoxicity and Enhances the Anticancer Effects of Sunitinib by Decreasing Pro-Oxidative Stress, Pro-Inflammatory Cytokines, and NLRP3 Inflammasome Expression

**DOI:** 10.3389/fonc.2021.680758

**Published:** 2021-06-11

**Authors:** Vincenzo Quagliariello, Massimiliano Berretta, Simona Buccolo, Martina Iovine, Andrea Paccone, Ernesta Cavalcanti, Rosaria Taibi, Monica Montopoli, Gerardo Botti, Nicola Maurea

**Affiliations:** ^1^ Division of Cardiology, Istituto Nazionale Tumori –IRCCS- Fondazione G. Pascale, Napoli, Italy; ^2^ Department of Clinical and Experimental Medicine, University of Messina, Messina, Italy; ^3^ Laboratory Medicine Unit, Istituto Nazionale Tumori- IRCCS-Fondazione G. Pascale, Napoli, Italy; ^4^ Department of Pharmacological Sciences, Gruppo Oncologico Ricercatori Italiani, GORI, Pordenone, Italy; ^5^ Department of Pharmaceutical and Pharmacological Sciences, Università degli Studi di Padova, Padova, Italy; ^6^ Scientific Direction, Istituto Nazionale Tumori- IRCCS- Fondazione G. Pascale, Napoli, Italy

**Keywords:** sunitinib, polydatin, cardio-oncology, microenvironment, chemokine

## Abstract

Renal cell carcinoma (RCC) represents the main renal tumors and are highly metastatic. Sunitinib, a recently-approved, multi-targeted Tyrosine Kinases Inhibitor (TKi), prolongs survival in patients with metastatic renal cell carcinoma and gastrointestinal stromal tumors, however a dose related cardiotoxicity was well described. Polydatin (3,4’,5-trihydroxystilbene-3-β-d-glucoside) is a monocrystalline compound isolated from Polygonum cuspidatum with consolidated anti-oxidant and anti-inflammatory properties, however no studies investigated on its putative cardioprotective and chemosensitizing properties during incubation with sunitinib. We investigated on the effects of polydatin on the oxidative stress, NLRP3 inflammasome and Myd88 expression, highlighting on the production of cytokines and chemokines (IL-1β, IL-6, IL-8, CXCL-12 and TGF-β) during treatment with sunitinib. Exposure of cardiomyocytes and cardiomyoblasts (AC-16 and H9C2 cell lines) and human renal adenocarcinoma cells (769‐P and A498) to polydatin combined to plasma-relevant concentrations of sunitinib reduces significantly iROS, MDA and LTB4 compared to only sunitinib-treated cells (P<0.001). In renal cancer cells and cardiomyocytes polydatin reduces expression of pro-inflammatory cytokines and chemokines involved in myocardial damages and chemoresistance and down-regulates the signaling pathway of NLRP3 inflammasome, MyD88 and NF-κB. Data of the present study, although *in vitro*, indicate that polydatin, besides reducing oxidative stress, reduces key chemokines involved in cancer cell survival, chemoresistance and cardiac damages of sunitinib through downregulation of NLRP3-MyD88 pathway, applying as a potential nutraceutical agent in preclinical studies of preventive cardio-oncology.

## Introduction

Tyrosine kinase-targeted therapies (TKi) have revolutionized the treatment of many cancers in the last years (1, 2). These drugs used alone or combined to other anticancer drugs have improved antitumor efficacy and have fewer toxic side-effects, compared to traditional chemotherapy, however, many adverse cardiac events have been recorded, including QT prolongation, heart failure and cardiac fibrosis (3, 4). Sunitinib malate (Sutent; Pfizer) is a multitargeted TKi that inhibits both tumor cell proliferation and angiogenesis in cancer tissue (5); it is approved to treat advanced renal cell carcinoma and gastrointestinal stromal tumors (5). However, patients treated with sunitinib have high incidence of hypertension, cardiac left ventricular systolic dysfunction and congestive heart failure (6, 7). The biochemical mechanism of sunitinib-induced cardiotoxicity is not well known but has been broadly attributed to the lack of kinase selectivity of sunitinib and to the intracellular targeting of mitochondrial proteins and oxidative stress (8). Similar to other TKis, the lack of selectivity of sunitinib makes it challenging to pinpoint whether there are specific molecular target(s) that are the critical mediators of cardiotoxicity (9). In addition to off-target kinase inhibition by sunitinib, AMP-activated protein kinase (AMPK) has been suggested to be a target of sunitinib-induced cardiotoxicity (10–12). The rhythmic con-traction of cardiac tissues requires a constant, stable source of energy, leaving a limited reserve of ATP. Thus, inhibition of AMPK mediated by sunitinib could leading to deleterious consequences including the activation of pro-apoptotic signals, cytochrome c release in cytosol and fibrosis (13).

Polydatin (trans-resveratrol-3-O-glucoside) is a natural compound belonging to the stilbenes class of the polyphenols (14). It is extracted from the roots of the *Poligonum Cuspidatum* plant, native to Asia but currently very widespread also in America and Europe (15). Polydatin is considered the “twin” molecule of resveratrol; both bioactives differ only in one glucose molecule, present in polydatin, a difference that makes it more soluble, more resistant to enzymatic attacks with a better oral bioavailability and pharmacokinetic profile compared to resveratrol (16, 17). Several studies indicates that polydatin is able to reduce the propagation (scavenger-scavenger effect) of reactive oxygen species, the production of nitric oxide and pro-inflammatory cytokines by inhibiting the NLRP3 inflammasome and the signaling of the NF-kB, both factors involved in cancer survival and cardiotoxic events like myocardial fibrosis, atherosclerosis and heart failure (18–20).

Polydatin is a well-established nutraceutical with anti-inflammatory properties (21, 22). As metabolite of resveratrol (23), pathways involved in polydatin-mediated beneficial effects involves also AMPK, NLRP3, MyD88 and prostaglandins homeostasis through reduction of iROS content in target cells (23, 24). Defined as complementary and alternative medicine (CAM), polydatin could have a great potential to became a natural cardioprotective agent in cancer patients (25). Here, we investigated on the putative cardioprotective and chemosensitizing effects of polydatin during incubation with sunitinib in cardiomyocytes and human renal adenocarcinoma cells highlighting on the underling pathways involved.

## Materials and Methods

### Cell Cultures and Treatments

Human cardiomyocytes (AC-16 cells) and cardiomyoblasts (H9C2 cells) were purchased from American Type Culture Collection (ATCC^®^, LGC Standards) and cultured in Gibco^®^ Dulbecco’s modified Eagle’s medium: Nutrient mixture F-12 (DMEM/F12) supplemented with 10% fetal bovine serum (FBS) (HyClone™, GE Healthcare Life Sciences) and Penicillin-Streptomycin (100 U/mL, Gibco^®^) in a humidified incubator at 37 °C with 5% CO_2_. Cultures were maintained in a humidified atmosphere of 95% air and 5% CO_2_ at 37°C. Human renal adenocarcinoma cells 769‐P and A498 were obtained from American Type Culture Collection (ATCC^®^, LGC Standards). A498 cells were cultured in DMEM, while 769‐P cells were cultured in RPMI. All media was supplemented with 10% fetal bovine serum (FBS) and 100 U/ml penicillin and 0.1 mg/ml streptomycin.

### Assessment of Cell Survival, Lactate Dehydrogenase, and Cytochrome C Release During Exposure to Sunitinib, Polydatin, or Both in Combination

To test the effects of sunitinib and polydatin on cellular mitochondrial viability, human cardiomyocytes and renal adenocarcinoma cells were plated in 96-well flat-bottom plates at the density of 150000 cells/well for 16 h. After three washes in PBS, cardiomyocytes and human renal cancer cells were untreated (control) or treated for 48 h with polydatin (Sig-ma Aldrich, Milan, Italy) at 50,100,200 and 400 µM or Sunitinib (sunitinib malate; SU11248; SUTENT; Pfizer Inc, New York, NY, USA) at 5,10,25 and 50 µM alone or combined to polydatin. After treatments, adherent cells were washed three times with PBS at pH 7.4 and incubated with 100 μL of an MTS solution (0.5 mg/mL in cell culture medium) for 4 h at 37°C, as described in literature (26). Absorbance readings were acquired at a wavelength of 450 nm with the Tecan Infinite M200 plate-reader (Tecan Life Sciences Home, Männedorf, Switzerland) using I-control software. Relative cell viability (%) was calculated with the following formula (A)test/(A)control × 100, where “(A)test” is the absorbance of the test sample, and “(A)control” is the absorbance of the control cells incubated solely in culture medium. After the evaluation of cell cytotoxicity, we measured the total protein content using the Pierce Micro BCA protein assay kit (Thermo Fisher, Milan, Italy) (27). Briefly, the cells were washed with ice-cold PBS, and incubated for 15 min in 150 μL cell lysis buffer (0.5% v/v Triton X-100 in PBS) that included 150 μL of the Micro BCA protein assay kit reagent (prepared according to the manufacturer’s instructions). Absorbance at 562 nm was measured on a plate reader. Cytotoxicity measurements were normalized by the amount of total protein content in each well. LDH release by damaged cells into the supernatant was determined by the Cytotoxicity Detection Kit (LDH) (Roche Applied Science) (28). Signals were quantified using a microplate spectrofluorometer at 490 nm for LDH quantification. For cytochrome c quantification in cell cytosol of cells, we incubated cells (5x107 cells/well) with cell media (control), sunitinib (10, 25µM), polydatin (100 and 200 µM) and both in combination for 48h. For cytochrome c extraction, cells were harvested and collected by centrifugation. After washing twice with ice-cold PBS, the cell pellet was treated according to the manufacturer’s protocol with the Cell Fractionation Kit (Clontech, Palo Alto, CA) (29). The cells were disrupted by douncing 60 times using a tissue grinder with a type A pestle on ice. After centrifugation at 700 g for 10 minutes, this supernatant was centrifuged at 10,000 g for 25 minutes to obtain the cytosol fraction. For cytochrome c quantification in cytosol fraction of the cells we through Human Cytochrome C ELISA Kit (BioTechne SRL, Milan, Italy) that provides an effective means for detecting cytochrome c translocation from mitochondria into cytosol (Sensitivity:0.31 ng/mL; Assay Range: 0.6 - 20 ng/mL)

### Measurement of Intracellular ATP Levels

Intracellular ATP contents were measured using ENLITEN^®^ ATP Assay System (Promega Italia S.r.l, Milan, Italy) according to the manufacturer’s instructions. Briefly, cardiomyocytes and renal cancer cells were untreated (control) or treated for 48 h with polydatin (100 and 200 µM) or Sunitinib (10 µM) alone or combined to polydatin. After treatments, 100 μl of lysis/assay solution provided by the manufacturer was added to confluent cell cultures in 96-well plates. After the plates were shaken for 1 min and incubated for 10 min at 23°C, luminescence was measured in a microplate luminometer (Thermo Fisher, Milan, Italy). Data were expressed as relative units (r.u) according to literature (30).

### Apoptosis Through Caspase−3/7 Activity Analysis

For the measurement of apoptosis, cardiomyocytes and renal cancer cells were untreated (control) or treated for 48 h with polydatin (100 and 200 µM) or Sunitinib (10 µM) alone or combined to polydatin and cultured with CaspaseGlo 3/7 reagent (Promega, Madison, USA) for half an hour at 37°C, according to literature (31). Caspase-3/7 activity was then determined by using a microplate spectrofluorometer.

### Expression of Leukotrienes B4

Cardiomyocytes and renal cancer cells were untreated (control) or treated for 12 h with polydatin (100 and 200 µM) or Sunitinib (10 µM) alone or combined to polydatin. After treatments, cells were lysed through triton X-100 (0,1% in PB) and leukotriene B4 [(5S,12R)-dihydroxy-6,14Z-8,10E-eicosatetraenoic acid] expression in cell lysates was determined through ELISA (Cayman Chemical) following the supplier’s instructions (32); data were expressed as pg of leukotriene B4/mg of cell proteins calculated by QuantiPro Assay (Biorad, Milan, Italy).

### Intracellular Reactive Oxygen Species and Lipid Peroxidation

Intracellular reactive oxygen species (iROS) and lipid peroxidation are key mediators of several cardiac dysfunctions induced by TKi and doxorubicin (33). Cardiomyocytes and renal cancer cells were untreated (control) or treated for 12 h with polydatin (100 and 200 µM) or Sunitinib (10 µM) alone or combined to polydatin. After treatments, cells were washed three times with cold PBS, harvested with 0.25% v/v Trypsin and centrifuged at 1000 ×g for 10 min. The supernatant was discarded and the cell pellet sonicated in cold PBS. After a centrifugation step at 800 ×g for 5 min, we quantified iROS and a marker of lipid peroxidation called malondialdehyde (MDA) by using a commercial kit with a spectrophotometer according to the manufacturer’s protocols (Sigma Aldrich, Milan, Italy) (34). We measured the protein content of the cell homogenates using the Micro BCA protein assay kit (Pierce, Thermo Fisher, Milan, Italy) according to kit instructions. Considering that sunitinib presents a green autofluorescence which can overlap with commercial probes used to measure iROS, data were normalized against sunitinib autofluorescence (control samples were constituted only by 0.25% v/v Trypsin in PBS + Sunitinib at 10 µM).

### Assessment of Protein Levels of NLRP3 Inflammasome, Myd88 (Myddosome) and NF-kB

Cardiomyocytes and renal cancer cells were untreated (control) or treated with polydatin (100 and 200 µM) or Sunitinib (10 µM) or both in combination for 12 h. After treatments, cells were harvested and lysed in lyses buffer (50 mM Tris-HCl, pH 7.4, 1 mM EDTA, 100 mM NaCl, 20 mM NaF, 3mM Na3VO4, 1mMPMSF, and protease inhibitor cocktail). Lysates were then centrifuged, the supernatants were collected and analyzed for quantification of NLRP3 inflammasome, Myd88 (myddosome) and NF-Kb. Quantification of MyD88 myddosome was performed through human and rat MyD88 ELISA Kit (ab171341), Abcam, Milan, Italy; NLRP3 inflammasome was quantified through Human and rat NLRP3 ELISA Kit (OKEH03368), Aviva Systems Biology, San Diego, CA, USA) (35–37). Briefly, an antibody against NLRP3 or MyD88 was pre-coated onto a 96-wellplate (12 × 8 Well Strips) and blocked. Standards or test samples were added to the wells and incubated for 1h. After washing, a biotinylated detector antibody specific to NLRP3 or MyD88 was added, incubated and followed by washing for 30 s. Avidin-Peroxidase Conjugate was then added, incubated, and unbound conjugate was washed away. An enzymatic reaction was produced through the addition of TMB substrate which is catalyzed by HRP generating a blue color product that changes yellow after adding acidic stop solution. The density of yellow coloration read by absorbance at 450 nm was quantitatively proportion-al to the amount of sample NLRP3 or MYD88 captured in well. For human MyD88 ELISA, the sensitivity was <10 pg/mL and range of detection was 156 pg/mL–10,000 pg/mL; for human NLRP3 ELISA assay, the sensitivity was <0.078 ng/mL and range of detection was 0.156–10 ng/mL. Analysis of NF-kB was performed through the use of TransAM NF-κB p65 transcription factor assay kit (Active Motif, Carlsbad, CA), according to the manufacturer’s recommendations (38). NF-κB complexes were captured by binding to a consensus 5′-GGGACTTTCC-3′ oligonucleotide immobilized on a 96-well plate. Bound NF-κB was quantified by incubating with anti-p65 primary antibody followed by horseradish peroxidase (HRP)-conjugated goat anti-rabbit IgG and spectrophotometric detection at a wavelength of 450 nm using a microplate spectrofluorometer. Data were expressed as the percentage of p65/NF-κB DNA binding relative to control (untreated) cells.

### Cytokines and Growth Factors Assay

The expression of IL-1β, IL-6, IL-8, CXCL-2, TGF-β and IL-18 in cardiomyocytes and human renal cancer cells was performed through ELISA method, as described elsewhere (39). Briefly, cells were exposed to polydatin (100 and 200 µM) or Sunitinib (10 µM) alone or combined to polydatin for 12 h. Culture supernatants were centrifuged to pellet any detached cells and measured using the appropriate ELISA kits according to the manufacturer’s instructions (Sigma Aldrich, Milan, Italy). The sensitivity of this method was below 10 (pg/mL), and the assay accurately detected cytokines in the range of 1–32000 pg/mL.

### NLRP3-IL-1β-IL-18 Pathways After Incubation With Resveratrol, the Natural Precursor of Polydatin

Polydatin is the natural bio derivate of resveratrol therefore, as control cardiomyocytes and renal cancer cells were unexposed (control) or exposed to resveratrol (Sigma Aldrich, Milan, Italy) (100 and 200 µM) or Sunitinib (10 µM) alone or combined to resveratrol for 12 h. After incubation period, expression of NLRP3 inflammasome, IL-1β and IL-18 were determined through ELISA method described before.

### Statistical Analysis

All cell-based assays were performed in triplicates and results are presented as mean ± Standard Deviation (SD). Statistical significance was analyzed by Student’s t test using Sigmaplot software (Systat Software Inc., San Jose, CA). p-value < 0.05 indicates a significant difference between two data values.

## Results

### Polydatin Enhances Survival of Cardiomyocytes and Increases Cytotoxicity in Renal Cancer Cells Exposed to Sunitinib

**Figure 1:**
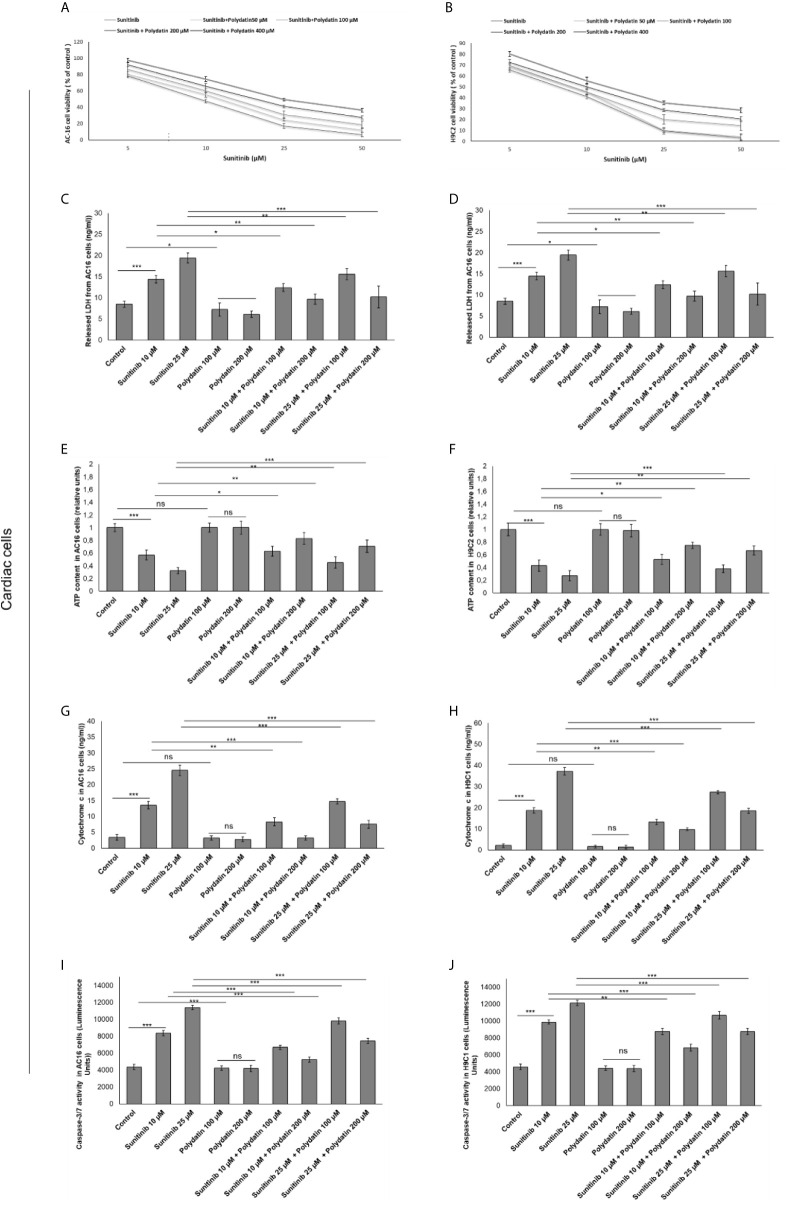
Cell viability **(A, B)**, cell death determined through LDH releases **(C, D)**, ATP content **(E, F)**, cytochrome c release **(G, H)** ad apoptosis assay thorough quantification od Caspase3/7 expression **(I, J)** in cardiac cells (AC16 and H9C2 cell lines) after 48h of incubation with sunitinib, polydatin or both in combination. Error bars depict means ± SD. p-values for the indicated compounds relative to untreated cells are: ***p<0.001. **p<0.01. *p<0.05. ns, not significant.

MTT assay was performed to investigate the effects of polydatin against the cytotoxic properties induced by sunitinib in cardiomyocytes and renal cancer cells (27, 28). The results showed that sunitinib decreased significantly the survival of cardiomyocytes in a concentration-dependent manner; for example, for AC16 cells the IC_50_ value was 11,5 ± 2,2 µM, in agree with other *in vitro* studies (**Figure 1A**). For this reason, the subsequent experiments (described from paragraph 2.1 to 2.5) were performed with a sunitinib concentration near to its IC_50_ value (10 µM). However, cytotoxicity results are slightly different from the literature (40, 41) where, as example, sunitinib reduces of 70% viability of H9c2 cells compared to control at 10 µM (difference of viability with previous reports should be due to the different origin of sunitinib). Similarly, LDH release was significantly increased after incubation with sunitinib (**Figures 1C, D**). When co-incubated with polydatin, cell viability was significantly increased in a concentration-dependent manner. Also in this case, cell death was determined by measuring the LDH released into the supernatant of sunitinib-treated cardiomyocytes cultures (29). The data showed that LDH release from cardiomyocytes decreased with increasing polydatin dose concentration up to 200 µM (P < 0,001) compared to sunitinib-treated cells (**Figures 1C, D**). Moreover, ATP content was significantly enhanced in cardiac cells (**Figures 1E, F**) co-exposed to sunitinib and polydatin compared to sunitinib group, confirming the cytoprotective properties of the natural compound. Notably, sunitinib exerts mitochondrial damages thereby increasing cytochrome c release, as reported in literature (29) (**Figures 1G, H**); polydatin significantly reduced its release from mitochondria, so improving mitochondrial homeostasis during exposure to sunitinib, reducing cell death (**Figures 1A, B**). Moreover, we studied apoptosis of cardiomyocytes through cellular quantification of Caspase3/7 expression (**Figures 1I, J**); as control, sunitinib induced apoptosis in cardiac cells by increasing Caspase 3/7 expression of around 45 and 57% (for 10 and 25 µM, respectively) compared to untreated cells (p<0.001 for both); polydatin reduces the magnitude of the effects confirming anti-apoptotic effects. These results indicated that polydatin significantly influenced the cytotoxicity of sunitinib in cardiomyocytes, reducing cell death.

**Figure 2:**
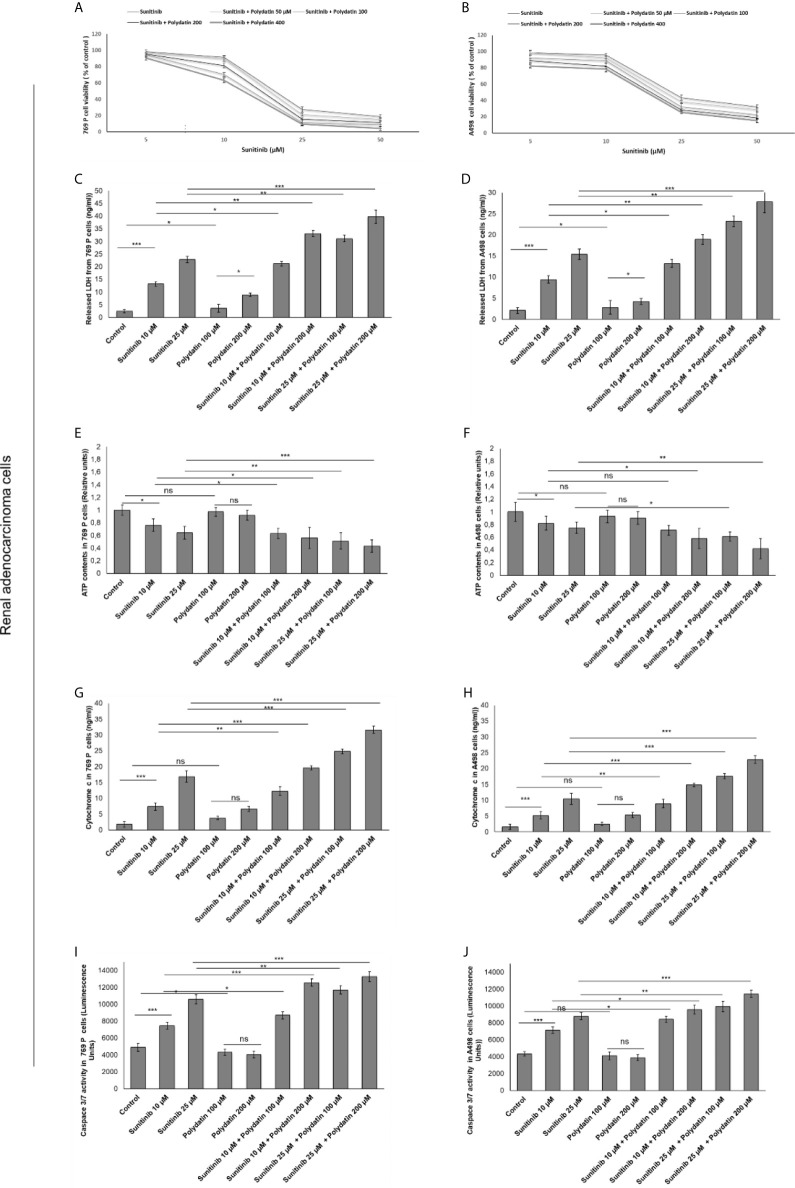
Cell viability **(A, B)**, cell death determined through LDH releases **(C, D)**, ATP content **(E, F)**, cytochrome c release **(G, H)** ad apoptosis assay thorough quantification od Caspase3/7 expression **(I, J)** in renal adenocarcinoma cells (769-P and A498 cell lines) after 48h of incubation with sunitinib, polydatin or both in combination. Error bars depict means ± SD. p-values for the indicated compounds relative to untreated cells are: ***p<0.001. **p<0.01. *p<0.05. ns, not significant.

In contrast, 769-P and A498 cells (**Figure 2**) showed a different behavior when co-exposed to polydatin and sunitinib compared to monotherapies. Firstly, polydatin slightly increased cell death in both renal cancer cells exposed to sunitinib alone (**Figures 2A, B**) increasing significantly both LDH (**Figures 2C, D**), ATP content (**Figures 2E, F**) and Cytochrome c release (**Figures 2G, H**) in a concentration dependent manner. Pro-apoptotic effects of polydatin against cancer cells were seen, with increased caspase3/7 expressions in combination therapies against sunitinib group (**Figures 2I, J**).

### Polydatin Reduces Leukotrienes B4 Levels During Exposure to Sunitinib

**Figure 3:**
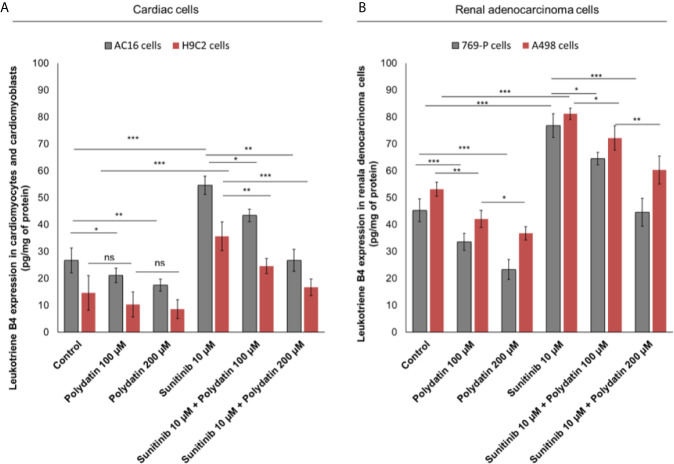
Leukotrienes type B4 production by AC-16 and H9C2 cells **(A)** or 769-P and A498 cells **(B)**, untreated (control) or treated with polydatin (100 and 200 µM) or sunitinib (10 µM) alone or combined to polydatin at 100 or 200 µM for 12h. Error bars depict means ± SD. p-values for the indicated compounds relative to untreated cells are: ***p<0.001. **p<0.01. *p<0.05. ns, not significant.

To evaluate the effects of polydatin on lipid metabolism transduction signal pathways during treatment with sunitinib, we quantified the production of leukotrienes B4 (LTB4) (**Figure 3**), convalidated as key players of 6-series prostaglandins involved in atherosclerosis, heart failure and cancer cell survival (32). Cardiomyocytes exposed to sunitinib increased the production of leukotrienes (For AC-16 cells, 54.6 ± 3.4 vs 26.7 ± 4.6pg/mg of protein, p<0.001) compared to untreated cells (**Figure 3A**); these effects were partially reduced in combination treatment with polydatin at 100 µM (For AC16 cells: 43.4 ± 2.3 pg/mg of protein) and 200µM (For AC16 cells: 26.7 ± 4.1 pg/mg of protein) (**Figure 3A**). A similar behavior was seen for renal adenocarcinoma cells (**Figure 3B**); cancer cells exposed to sunitinib increased the production of leukotrienes (For 769-P cells, 76.8 ± 4.4 vs 45.3 ± 4.2 pg/mg of protein, p<0.001) compared to untreated cells (**Figure 3B**); these effects were partially reduced in combination treatment with polydatin at 100 µM (For 769-P cells: 64.5 ± 2.1 pg/mg of protein) and 200µM (769-P cells: 44.6 ± 5.2 pg/mg of protein) (**Figure 3B**).

### Polydatin Reduces Intracellular Reactive Oxygen Species and MDA Production During Exposure to Sunitinib

**Figure 4:**
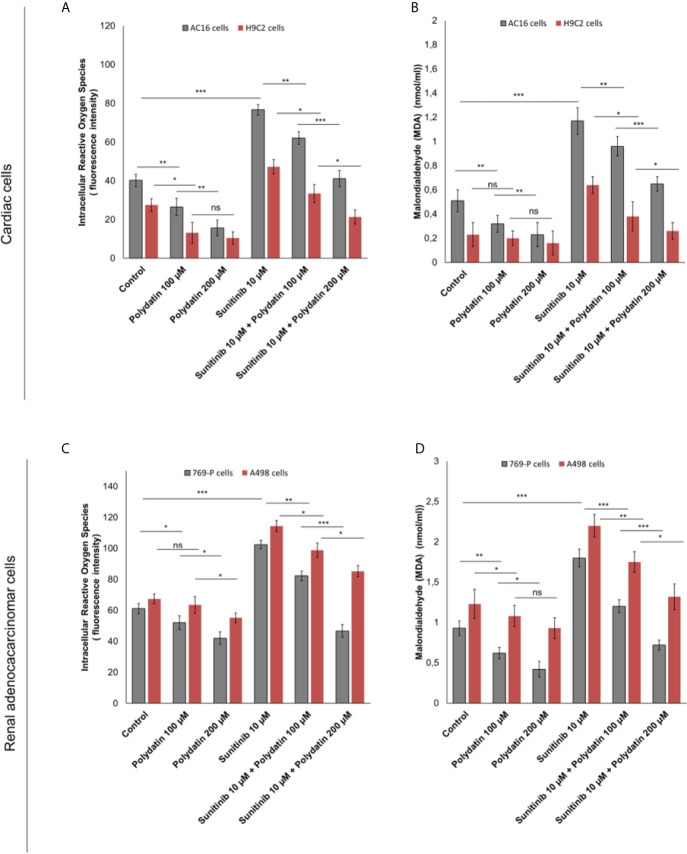
**(A, C)** Intracellular Reactive Oxygen Species (iROS) and **(B, D)** Malondialdeyde (MDA) quantification in cardiac cells (AC-16 and H9C2 cell lines) or human adenocarcinoma cells (769-P and A498 cell lines) untreated (control) or treated with polydatin (100 and 200 µM) or sunitinib (10 µM) alone or combined to polydatin at 100 or 200 µM for 12h. Error bars depict means ± SD. p-values for the indicated compounds relative to untreated cells are: ***p<0.001. **p<0.01. *p<0.05. ns, not significant.

Intracellular reactive oxygen species (iROS) are involved in chemoresistance to TKi and in cardiotoxicity mediated by several anticancer drugs like daunorubicin, cisplatin, doxorubicin, 5-fluorouracil and alkylating agents (34, 35). A recent cellular study indicated that TKi (including sunitinib) induced cardiomyocyte cell death through production of iROS (34). In cardiac cells, treatment with sunitinib increased iROS production (**Figure 4A**); polydatin drastically reduced the magnitude of the effects in a concentration-dependent manner (For AC16 cells, 20.3% and 45,2% for 100 and 200 µM, respectively compared to only sunitinib treated cells; p>0,001). These effects were confirmed through the quantification of malondialdeyde (MDA) as marker of lipid peroxidation (34) that was increased significantly during incubation with sunitinib (For AC16 cells, 1.17 ± 0.11 mmol/ml vs 0.51 ± 0.09 mmol/ml for untreated cells, p<0.001) (**Figure 4B**) and reduced in combination treatment with polydatin (For AC-16 cells 0.96 ± 0.08 mmol/ml and 0.65 ± 0.06 mmol/ml for polydatin 100 and 200 µM, respectively). In renal adenocarcinoma cells, treatment with sunitinib increased iROS production (**Figure 4C**); polydatin drastically reduced the magnitude of the effects in a concentration-dependent manner (For 769-P cells, 13.7% and 38,7% for 100 and 200 µM, respectively compared to only sunitinib treated cells; p> 0,001). Also malondialdeyde (MDA) was increased significantly during incubation with sunitinib (For 769-P cells, 1.83 ± 0.13 mmol/ml vs 0.93 ± 0.18 mmol/ml for untreated cells, p<0.001) (**Figure 4D**) and reduced in combination treatment with polydatin (For 769-P cells 1.2 ± 0.13 mmol/ml and 0.72 ± 0.09 mmol/ml for polydatin 100 and 200 µM, respectively).

### Polydatin Reduces NLRP3 Inflammasome, Myd88 Myddosome, and NF-kB Expression During Treatment With Sunitinib

**Figure 5:**
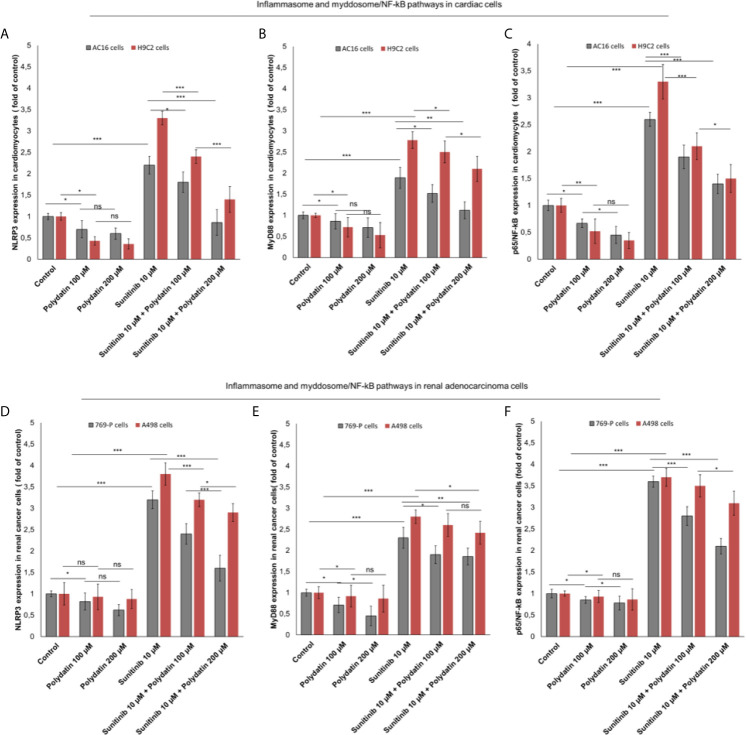
**(A, D)** NLRP3 inflammasome **(B, E)** Myd88 myddosome **(C, F)** and p65/NF-kB (fold of control) expression in cardiac cells (AC-16 and H9C2 cell lines) and renal adenocarcinoma cells (769-P and A498 cell lines) untreated (control) or treated with polydatin (100 and 200 µM) or sunitinib (10 µM) alone or combined to polydatin at 100 or 200 µM. Error bars depict means ± SD. p-values for the indicated compounds relative to untreated cells are: ***p<0.001. **p<0.01. *p<0.05. ns, not significant.

NLRP3 inflammasome is the key player of cytokine storm involved in cancerogenesis, heart failure and atherosclerosis (37, 38). Recently, our group investigated on the role of NLRP3 in cardiotoxicity of immune check-point inhibitors (39). Effectively, as example, in AC16 cells (**Figure 5A**). NLRP3 is also involved in sunitinib-mediated cell death; in fact, its expression is clearly enhanced after exposure to sunitinib compared to untreated cells (2.2 ± 0.21 vs 1 ± 0.07 (fold of control) p<0.001); lower levels of NLRP3 after co-incubation with polydatin were seen (1.8 ± 0.24 and 0.86 ± 0.3 for 100 and 200 µM, as fold of control) p<0.001 (**Figure 5A**). A similar behavior was seen for MyD88 myddosome, another macromolecular complex involved in heart failure and cardiac fibrosis after exposure to viral and chemical agents with cardiotoxic properties. In fact, Myd88 expression is clearly enhanced after exposure to sunitinib compared to untreated cells (1.89 ± 0.25 vs 1 ± 0.08, (fold of control) p<0.001); lower levels of Myd88 after co-incubation with polydatin were also seen (1.52 ± 0.21 and 1.12 ± 0.2 for 100 and 200 µM, as fold of control) p<0.001 (**Figure 5B**). A similar behavior was seen in renal adenocarcinoma cells (**Figures 5D, E**); NLRP3 was overexpressed after exposure to sunitinib compared to untreated cells (3.3 ± 0.26 vs 1 ± 0.23, (fold of control) p<0.001); lower levels of NLRP3 after co-incubation with polydatin were seen (2.4 ± 0.16 and 1.6 ± 0.21 for 100 and 200 µM, as fold of control) p<0.001 (**Figure 5D**). Similarly, Myd88 expression was enhanced after exposure to sunitinib compared to untreated cancer cells (2.33 ± 0.16 vs 1 ± 0.14, (fold of control) p<0.001); lower levels of Myd88 after co-incubation with polydatin were also seen (1.91 ± 0.27 and 1.86 ± 0.27 for 100 and 200 µM, as fold of control) p<0.001 vs sunitinib-exposed cells (**Figure 5E**). Considering that NLRP3 inflammasome and myddosome are the most important activators of NF-kB in human cells, we investigated on its expression during exposure to sunitinib and polydatin. As shown in **Figures 5C, F**, NF-κB expression was significantly increase of 2.6 and 3.46 times in AC-16 and 769-P cells, respectively, exposed to sunitinib at 10 µM, in agree with other *in vitro* studies recently published (40, 41). Additionally, cardiomyocytes and renal cancer cells exposed to sunitinib and polydatin partially reduced expression of NF-κB (**Figures 5C, F**). These effects indicate anti-inflammatory properties of polydatin during treatment with sunitinib in cardiac and renal adenocarcinoma cells.

### Polydatin Reduces Cytokines and Growth Factors Involved in Cardiac Dysfunction and Chemoresistance to Sunitinib

**Figure 6:**
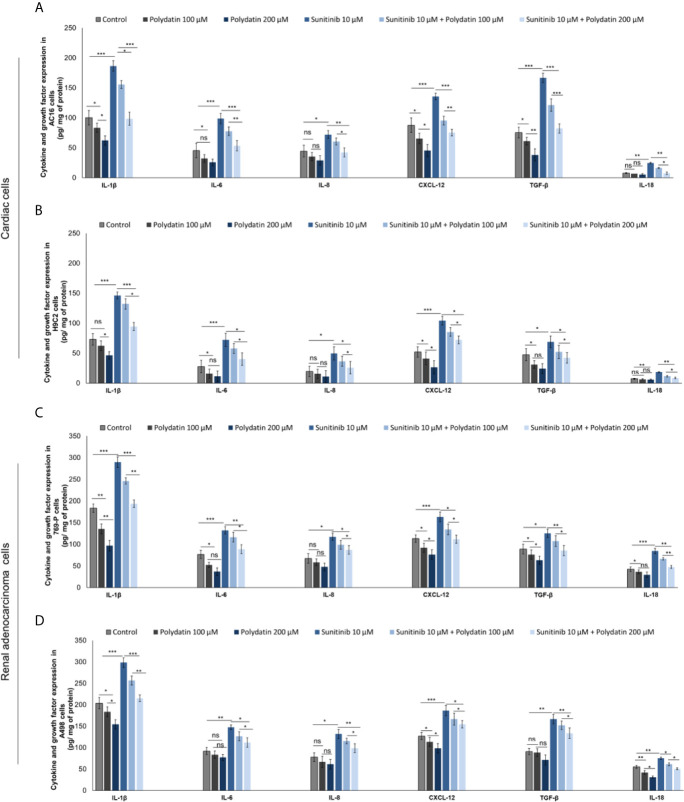
Expression of IL-1β, IL-6, IL-8, CXCL-12, TGF-β and IL-18 in cardiac cells **(A, B)** (AC-16 and H9C2 cells) and renal adenocarcinoma cells **(C, D)** (769-P and A498 cell lines); cells were untreated (control) or treated with polydatin (100 and 200 µM) or sunitinib (10 µM) alone or combined to polydatin at 100 or 200 µM. Error bars depict means ± SD. p-values for the indicated compounds relative to untreated cells are: ***p<0.001. **p<0.01. *p<0.05. ns, not significant.

As well know, hyper activation of NF-kB, NLRP3 and MyD88 increases the production of cytokines involved in antiviral and anticancer response as well as in cardiotoxic events (42–47). We investigated on the production of cytokines and growth factors by cardiac cells and renal cancer cells during exposure to sunitinib alone or combined to polydatin. Firstly, AC-16 cells exposed to sunitinib (**Figures 6A, B**) overexpressed IL-1β (186.5 ± 8.8 vs 100.2 ± 12.3 pg/mg of protein, p<0.001), IL-6 (98.7 ± 8.6 vs 45.5 ± 9.9 pg/mg of protein, p<0.001), IL-8 (72.1 ± 7.7 vs 44.5 ± 9.8 pg/mg of protein, p<0.001), CXCL-12 (135.5 ± 5.5 vs 87.6 ± 12.2 pg/mg of protein, p<0.001) and TGF-β (166.5 ± 10.2 vs 75.5 ± 8.9 pg/mg of protein, p<0.001), than untreated cells. After co-incubation with polydatin, the rates of increase of cytokines and growth factors were significantly reduced, indicating anti-inflammatory effects of the nutraceutical compound, in agree with other published works (48, 49). For example, co-incubation with sunitinib and polydatin at 100 µM decreased significantly the expression of all cytokines involved in cell death and cardiac fibrosis compared to sunitinib-treated cells: IL-1β (186.5 ± 8.8 vs 155.6 ± 6.5 pg/mg of protein, p<0.05), IL-6 (77.6 ± 7.2 vs 98.7 ± 8.6 pg/mg of protein, p<0.01), IL-8 (60.5 ± 5.6 vs 72.1 ± 7.7 pg/mg of protein, p<0.05), CXCL-12 (95.5 ± 7.3 vs 135.5 ± 5.5 pg/mg of protein, p<0.01), TGF-β (121.2 ± 10.5 vs 166.5 ± 10.2 pg/mg of protein, p<0.001) and IL-18 (16.3 ± 0.8 vs 24.6 ± 1.1 pg/mg of protein, p<0.05). A similar behavior was seen for cardiomyoblasts H9C2 cells (**Figure 6B**). Renal adenocarcinoma cells (**Figures 6C, D**) exposed to sunitinib increased the production of all cytokines involved in cancer cell survival and chemo resistance. For example, 769-P cells (**Figure 6C**) exposed to sunitinib overproduced IL-1β (289.4 ± 9.6 vs 183.2 ± 8.5 pg/mg of protein, p<0.001), IL-6 (132.1 ± 8.3 vs 76.5 ± 9.4 pg/mg of protein, p<0.001), IL-8 (117.4 ± 8.9 vs 67.4 ± 11.2 pg/mg of protein, p<0.001), CXCL-12 (163.2 ± 15.1 vs 113.2 ± 8.7 pg/mg of protein, p<0.001), TGF-β (125.3 ± 9.6 vs 88.9 ± 11.3 pg/mg of protein, p<0.001) and IL-18 (66.3 ± 2.7 vs 84.5 ± 4.3 pg/mg of protein, p<0.05) than untreated cells. Co-incubation with polydatin reduced the magnitude of the effects in a significant manner. These results indicated that polydatin change cardiac and renal cancer microenvironment through a significant reduction of IL-1β, IL-6, IL-8, CXCL-12, TGF-β and IL-18.

### Resveratrol, the Natural Precursor of Polydatin, Reduces NLRP3 Inflammasome-IL-1β-IL-18 Pathways During Exposure to Sunitinib

**Figure 7:**
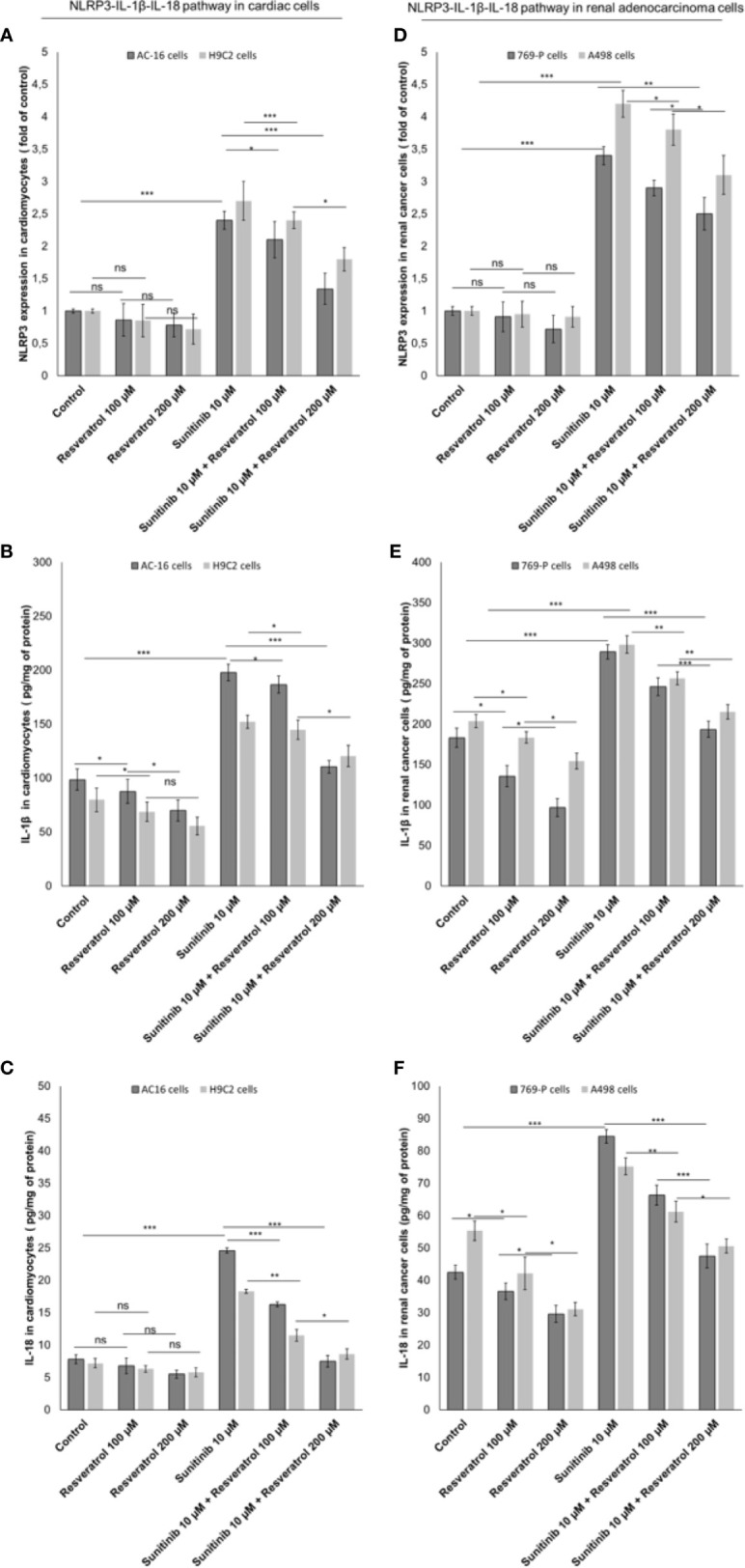
Expression of NLRP3 **(A, B)**, IL-1β **(C, D)** and IL-18 **(E, F)** in cardiac cells (AC-16 and H9C2 cells) and renal adenocarcinoma cells (769-P and A498 cell lines); cells were untreated (control) or treated with resveratrol (100 and 200 µM) or sunitinib (10 µM) alone or combined to resveratrol at 100 or 200 µM. Error bars depict means ± SD. p-values for the indicated compounds relative to untreated cells are: ***p<0.001. **p<0.01. *p<0.05. ns, not significant.

Resveratrol is the natural precursor of polydatin (50). As control we investigated on the NLRP3 expression and production of IL-1β and IL-18 by cardiac cells and renal cancer cells during exposure to sunitinib alone or combined to resveratrol. As show in **Figure 7**, resveratrol was able to reduce significantly the expression of pro-inflammatory biomarkers both in cancer cells and cardiomyocytes exposed to Sunitinib. Specifically, resveratrol at 100 µM associated to sunitinib reduces of 15.4% the expression of NLRP3 inflammasome compared to sunitinib group (p<0.05) in AC-16 cells; IL-1β and IL-18 expressions were also reduced compared to sunitinib alone (182.3 ± 7.4 vs 198.8 ± 7.7 pg/mg of protein, p<0.05; 16.3 ± 0.26 vs 24.6 ± 0.4 pg/mg of protein, p<0.05; respectively). A similar behavior was seen for renal adenocarcinoma cells. These results confirms that resveratrol was able to reduce NLRP3 inflammasome, IL-1β and IL-18 expression similarly to polydatin during exposure to sunitinib indicating anti-inflammatory effects (**Figure 7**).

## Discussion

In this study we demonstrated that polydatin reduced cardiotoxicity and increases anticancer properties of Sunitinib in cellular models through the involvement of iROS, leukotrienes, MyD88 and NLRP3 signaling pathways. More specifically, our findings provide a proof of principle that polydatin reduced cytokine storm in cardiomyocytes and renal cancer cells thereby modulating their survival during exposure to sunitinib. Sunitinib is a tyrosine kinase inhibitor used in the treatment of renal cell carcinoma, gastrointestinal stromal and colorectal cancers (51–53). Sunitinib blocks cell signaling by targeting the adenosine-5′-triphosphate (ATP) binding sites of multiple receptor tyrosine kinases (54), overexpressed in cancer cells but normally expressed in non-cancer tissues like endothelial cells and heart (55). Tyrosin kinases play important roles in angiogenesis and tumor cell proliferation and are receptors for platelet-derived growth factor (PDGF) and vascular endothelial growth factor (VEGF) (56, 57). The attenuation of vascularization leads to apoptosis (58). However, Sunitinib lacks tyrosine kinase selectivity and results cardiotoxicity (59). Specifically, Sunitinib is a potent inhibitor of VEGF-1, VEGF-2, fetal liver tyrosine kinase receptor 3 (FLT3), KIT (stem-cell factor (SCF) receptor), PDGF-α, and PDGF-β (55). Patients treated with Sunitinib experienced asymptomatic QT prolongation, acute coronary syndrome, myocardial infarction, and symptomatic congestive heart failure (60). Recent real-world experiences and pivotal trials reports the lethality of sunitinib based on the adverse events data between 2-4%, indicating a clinically relevant toxicity that requires innovative cardioprotection strategies aimed to improve overall survival (61, 62). Interestingly, several circulating biomarkers like cytokines, are investigated in RCC patients (63), including IL-6, IL-1 and others; in fact, in RCC, pretreatment concentrations of plasma biomarkers (e.g., cytokines and angiogenic factors) have previously been studied in order to predict the outcome of VEGF or mTOR blocking agents (64).

Known mechanisms of sunitinib-induced cardiotoxicity involves the inhibition of cAMP-activated protein kinase (PK) which is crucial for post-hypoxia cell survival. Thus, sunitinib may induce cardiomyocyte hypertrophy and death (65, 66). Therefore, Sunitinib is an useful antineoplastic agent but may cause cardiomyopathy, left ventricular dysfunction, and heart failure. The adverse effects of Sunitinib may be attributed to its initiation of mitochondrial dysfunction (67). Mitochondrial dysfunction and redox imbalance may contribute to pathological states known as “free radical diseases” (68). The findings of the present study align with those of earlier reports. The administration of Sunitinib significantly decreased GSH and GR activity and a significant increased MDA content in cardiac tissues (69). Sunitinib is toxic against cardiac fibroblasts due to iROS generation (70). Earlier research demonstrated that sunitinib is cardiotoxic and may induce iROS in cardiac cells (71); in fact, it directly affects various cell membranes and may upregulate the gene encoding iNOS (72). Other reports established that sunitinib-induced toxicity is associated with the mRNA expressions of the TGF-β and smad-2/3 signaling molecules (73, 74). Moreover, with upcoming improved imaging technologies more and more cardiac metastases are seen in RCC patients (75, 76), therefore, a strictly prevention, diagnosis and management of cardiovascular complications in these patients should be performed, especially in combination therapies involving different TKi or association of TKi and ICIs (77, 78); moreover, cardioprotective and anticancer strategies aimed to target cardiac tissues should be investigated in preclinical trials.

Complementary and alternative medicines (CAM) are natural bioactives with anti-inflammatory, anticancer and chemo preventive properties. Several clinical trials demonstrated the safe and useful of CAM in cancer patients (79–82). Polydatin, also called piceide, is a metabolite of trans-resveratrol, a natural bioactive isolated from the perennial herb *Polygonum cuspidatum* (83), with interesting properties in the field of cancer, cardiovascular diseases, neurodegenerative disorders (84, 85). A recent study demonstrated that polydatin reduces tissue damages induced by hyperglycemia and hyperlipidemia, increasing cardiovascular functions during diabetes (86, 87). In another preclinical trial, 8-weeks polydatin treatment significantly improved the diastolic and systolic dysfunction, attenuated hypertrophy and interstitial fibrosis in mice with diabetes (88). Other similar studies suggested that polydatin protects cardiomyocytes heart failure also in non-diabetic models through reduction of iROS, pro-inflammatory prostaglandins and cytokines (89, 90).

**Figure 8:**
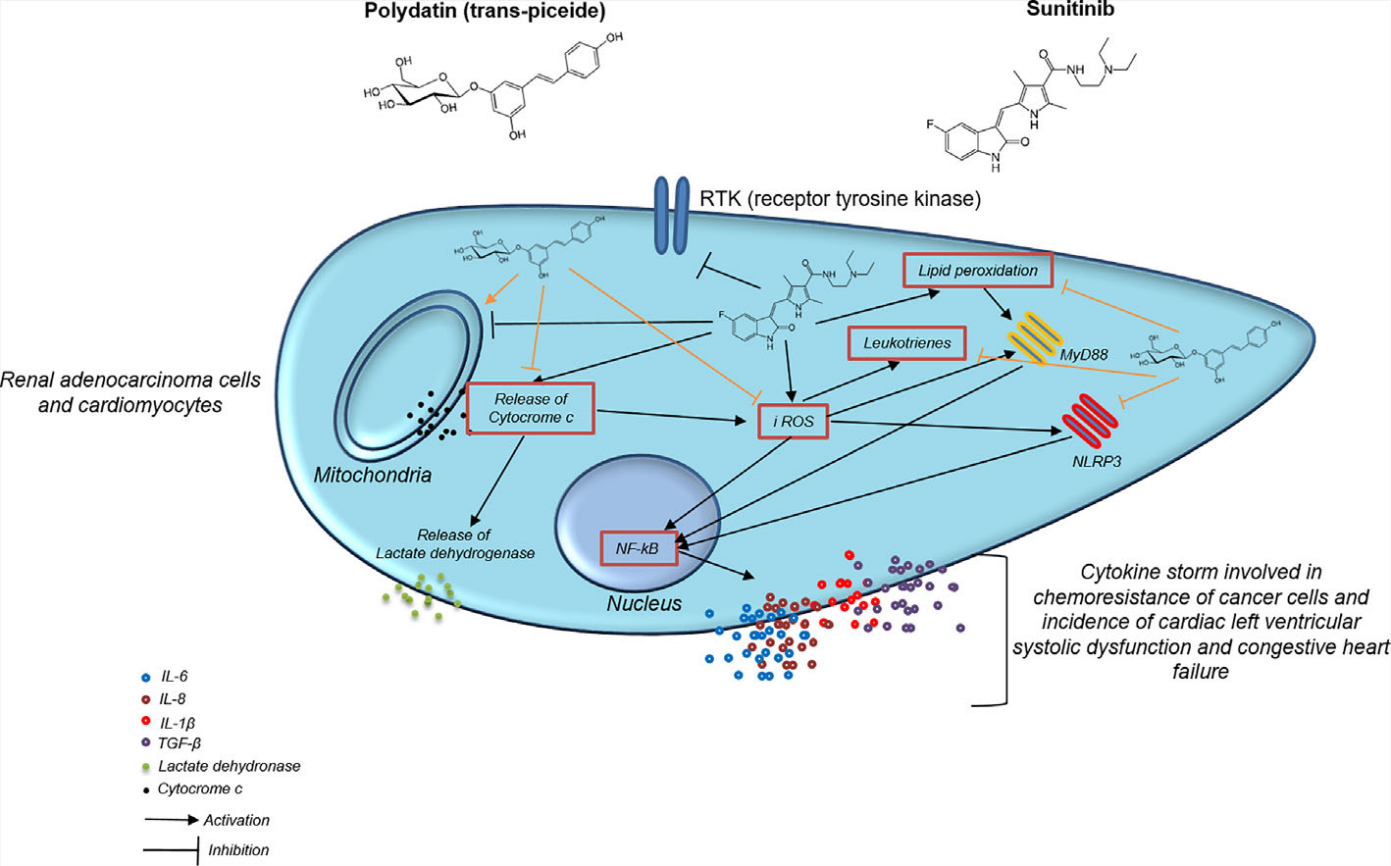
Mechanistic illustration of chemosensitizing and cardioprotective properties of polydatin during exposure to the TKi sunitinib.

As summarized in **Figure 8**, in the present study, sunitinib upregulated TGF-β, IL-1, IL6, IL-8 and CXCL12 both in cardiac cells and human renal adenocarcinoma cells that are involved in cell death and heart failure as well as in cancer cell survival and resistance to apoptosis (91, 92). Cytokine storm involved in heart failure, myocardial fibrosis, atherosclerosis and cardiotoxicity induced by anticancer drugs is induced by overexpression of MyD88 and NLRP3 inflammasome (93, 94). Myd88 is a molecular complex involved in regulation of cardiovascular and cancer metabolism as well as in cancer cell survival and chemo resistance (95, 96). Patients with myocarditis have high heart expression of MyD88, CD3+ lymphocytes and collagen fibers and these phenomena was recently seen in patients with SARS-CoV-2 infection (97). Inflammasomes are molecular complexes regulating pro-inflammatory factors, including IL-1β and IL-6 (98). IL-6 induces programmed cell death protein 1-dependent immunosuppression in cancer (99), and IL-1β is one of the most important pro-inflammatory mediators involved in chemo-resistance (100). NOD-like receptor family pyrin domain–containing 3 (NLRP3) is the most widely studied inflammasome (26). A recent study demonstrated that activated NLRP3 significantly promoted the plasma levels of cytokines in patients with cancer (101). Notably, NLRP3 inflammasome represents a novel potential target for the treatment of breast cancer (102). A recent preclinical study has clearly shown that inhibition of NLRP3 by miRNA is able to block tumor growth and the immune-resistance of breast cancer through ASC/IL-1/IL-18 pathways; this inhibition provides new clinical insights for the therapy of breast cancer (103). Notably, NLRP3 is associated to myocardial injuries, atherosclerosis and diabetes mellitus (104); Sunitinib, through the induction of iROS and lipid peroxidation, activated MyD88 and NLRP3 increasing inflammation and pro-fibrotic state in cultured cells; notably, treatment with polydatin combined to sunitinib reduced the magnitude of these effects as well as the expression of MyD88 and NLRP3 in a concentration dependent fashion. Myddosome and inflammasome activates expression and release of cytokines, chemokines and growth factors involved in cell death, fibrosis, chemoresistance and apoptosis (105). IL-1β, IL-6, IL-8 and IL-18 reduces mitochondrial metabolism, calcium homeostasis and viability of cardiomyocytes, fibroblasts and pericardial cells (106, 107). Recently, selective inhibitors of IL-1β, IL-6 and IL-8 are proposed in cancer patients aimed to improve anticancer effects of selective inhibitors of kinases and immune check-point inhibitors, reducing their toxicity (108, 109).

We demonstrated that sunitinib increased IL-1β, IL-6,IL-8 and IL-18 expression in cardiomyocytes and renal adenocarcinoma cells and that polydatin is able to significantly reduce their expression. In conclusion, previous studies have investigated the mechanisms of cardiotoxicity mediated by sunitinib indicating that it might promotes endothelial dysfunction, myocytes death and apoptosis through the disruption of mitochondrial homeostasis (110). However, its effects on NLRP3, Myd88 and pro-inflammatory cytokines involved in cardiotoxicity was never completely demonstrated. The present study, with all potential limitations derived from an ‘‘*in vitro*’’ investigation, points out for the first time that polydatin reduces iROS production and lipid peroxidation as well as the expression of pro-inflammatory chemokines through MyD88 and NLRP3 mediated pathways; based on the results obtained, further *in vivo* studies in renal cancer-bearing mice treated with sunitinib are suggested.

## Data Availability Statement

The raw data supporting the conclusions of this article will be made available by the authors, without undue reservation.

## Author Contributions

Conceptualization, VQ, MB, and NM. Methodology, VQ, SB, MI, and AP. Software, EC and RT. Validation, GB and NM. Formal analysis, SB, MI, and MB. Investigation, writing—original draft preparation, VQ. writing—review and editing, NM, GB and MB. Visualization: RT, NM, MB, and GB. Supervision, NM and GB. Funding acquisition, GB and NM. All authors contributed to the article and approved the submitted version.

## Funding

This work was funded by an “Ricerca Corrente” grant from the Italian Ministry of Health. Cardiotossicitá dei trattamenti antineoplastici: identificazione precoce e strategie di cardioprotezione” Project code: M1/5-C.

## Conflict of Interest

The authors declare that the research was conducted in the absence of any commercial or financial relationships that could be construed as a potential conflict of interest.
